# Clinical Study of Advanced Glycation End Products in Egyptian Diabetic Obese and Non-Obese Patients

**Published:** 2011-09

**Authors:** Mohamed N. Amin, Amany A. Mosa, Mamdouh M. El-Shishtawy

**Affiliations:** 1 *Department of Biochemistry, Faculty of Pharmacy, Mansoura University, Mansoura, Egypt;*; 2 *Department of Internal Medicine, Specialized Medical Hospital, Faculty of Medicine, Mansoura University, Mansoura, Egypt*

**Keywords:** advanced glycation end products, carboxymethyllysine, receptors for advanced glycation end products, obesity and type 2 diabetes mellitus

## Abstract

Advanced glycation end products (AGEs) are complex, heterogenous molecules generated by glycation and oxidation of proteins *in vivo*, which are thought to markedly increase in diabetic patients. One of the recently identified AGEs is carboxy methyl lysine (CML), which is the main ligand of receptors for advanced glycation end products (RAGE). The present study aimed to assess the effect of obesity on such pathways in presence and absence of Type 2 diabetes mellitus. CML, soluble receptors for advanced glycation end products (sRAGE), Hb_A1C_, lipid profile, liver function tests and kidney function tests were determined in 29 diabetic obese, 29 diabetic non-obese, 15 non-diabetic obese and 15 non-diabetic non-obese subjects. The study compared obese and non-obese subjects in presence and absence of type 2 diabetes. The results showed a significant increase in CML and a significant decrease in sRAGE in each of the diabetic obese group when compared with the diabetic non-obese group and the non-diabetic obese group when compared with the non-diabetic non-obese group. A significant positive correlation was found between CML and markers of obesity (body mass index and waist/hip ratio). These results suggest that obesity can increase CML independent of diabetes and support the reports that CML could be generated from both sugars and lipids. The present study suggests that treatment using glycation inhibitors like aminoguanidine or recombinant sRAGE will not only retard the diabetic complications, but may also have a prophylactic effect.

## INTRODUCTION

AGEs are heterogeneous compounds including compounds derived from protein glycation and their precursors. Protein glycation (Maillard reaction) occurs between reducing sugars and free amino-groups of a protein via nucleophilic addition that forms a Schiff base (1). The formation of reactive α-dicarbonyls occurs due to several rearrangements of Schiff base. The α-dicarbonyls can react with lysine and arginine functional groups on proteins, leading to the formation of stable AGE compounds, such as N-ε-(carboxymethyl)lysine adducts (CML).

CML can originate from both sugar and lipid oxidation products and were called either advanced glycation or lipoxidation end products (2). Consequently, CML is thought to be a general marker of oxidative stress (3). One of the main downstream pathways of CML is activation of the receptors of advanced glycation end products (RAGE). RAGE is a multiligand member of the immunoglobulin super-family of cell-surface molecules.

RAGE was first described as receptor for adducts modified by non-enzymatic glycosylation occurring on proteins and lipids in a wide variety of settings, mainly in diabetes mellitus (4). The best documented consequences of RAGE activation is the generation of reactive oxygen species, activation and translocation of pro-inflammatory kinases and transcription factors like nuclear factor kappa B (NFκB) (5).

AGEs are able to alter physiological processes *in vivo* by different mechanisms. AGE-mediated cross-linked proteins have decreased solubility and high resistance to proteolytic digestion, with the consequence of altered physicochemical and mechanical properties of tissue components (e.g. increased stiffness of collagen) and the generation of new immunogenic epitopes. Also, the binding of AGEs on the RAGE induces activation of NFκB of the RAGE bearing cells, resulting in increased expression of cytokines, growth factors and adhesion molecules. In addition, the accumulation of AGEs intensifies the expression of RAGE. RAGE is not only a receptor for AGEs but also for other pro-inflammatory molecules such as high-mobility group protein B1 and the S 100/calgranulin family, possibly increasing inflammatory reactions by this indirect way (6). On the other hand, blockade of AGE-mediated effects was observed in the presence sRAGE (7). sRAGE has been suggested to act as a decoy that captures the circulating AGEs preventing activation of RAGE signaling pathway (1).

The present study aimed to evaluate the effect of obesity on markers of the non-enzymatic glycation pathway (CML, sRAGE and HbA1C) and the contribution of type 2 diabetes. In addition, we investigated their diagnostic value for type 2 diabetes and obesity. *In vivo* studies of CML in obesity are scarce. Advanced glycation has been well studied in diabetes but studies have determined CML *in vivo* in obesity are still scarce. We tried to explore such unrevealed area to find out CML levels in obesity and type 2 diabetes. The study also aimed to investigate the origin of CML and the decoy function of sRAGE.

## SUBJECTS AND METHODS

### Subjects

The present study was carried out on 73 patients selected from the outpatients of the Diabetes Clinics and the Obesity Clinics of the Specialized Internal Medicine Hospital, Mansoura University, Egypt, between May 2009 and November 2009. Patients were divided into 29 Diabetic Obese group (D.O), 29 Diabetic Non-Obese group (D.N.O) and 15 Non-Diabetic Obese group (N.D.O). 15 Non-Diabetic Non-Obese subjects of Mansoura City inhabitants were selected as control group (N.D.N.O).

All patients and controls were subjected to history taking, thorough clinical examination, and blood pressure measurement. The exclusion criteria included: altered hepatic function (either by clinical examination or liver function tests), chronic inflammatory diseases, malignancy, and smoking. Body weight (to the nearest kg) and body height (to the nearest cm) were measured to calculate the body mass index (BMI). Obesity was defined by BMI more than 30 kg/m^2^ (8). The waist/hip ratio (WHR) was measured as a marker for abdominal obesity (9).

### Blood sampling

The selected patients and control subjects were fasted for 12 hours. Blood samples were collected by clean venipuncture and divided into two portions. 1.5 ml was collected into EDTA powder-containing wassermann tube, stored at 2-8°C and used for determination of blood glycated hemoglobin % (Hb_A1C_) using ion-exchange resin separation method (10). 3.5 ml was collected into clean dry wassermann tube, allowed to clot for 15 min at room temperature and centrifuged for 10 min at 600 g. The clear non-hemolyzed serum was used for the determination of the levels of fasting blood glucose (FBG) (11), total cholesterol (Total-C) (12), triglycerides (TAG) (13), high density lipoprotein-cholesterol (HDL-C) (14), alanine aminotransferase activity (ALT) (15), aspartate aminotransferase activity (AST) (16), albumin (ALB) (17) and creatinine (CRT) (18), immediately. Low density lipoprotein-cholesterol (LDL-C) was calculated from HDL-C, TAG and Total-C (19). The rest of serum was kept frozen at -20°C for the determination of CML using commercially available ELISA kit (Uscn Lifescience & Technology Co., Ltd, U.S.A) (20) and sRAGE concentration using commercially available ELISA kit (R&D Systems, Inc., U.S.A) (21). The ELx800 Absorbance Microplate ELISA Reader from BioTek Instruments, U.S.A with BioTek’s Gen5™ Data Analysis Software was used for ELISA measurements.

### Statistical analysis

Data were expressed as mean ± standard deviation (S.D). One way ANOVA test was used to compare between groups, followed by post hoc test (least significant difference) for inter-group comparisons. For correlation study, Pearson correlation was applied. Statistical calculations were done using the computer software SPSS version 10 (Chicago, IL, USA) and Microsoft Excel (Microsoft^®^OfficeExcel^®^2007). Statistical significance was set at *P*<0.05.

## RESULTS

### Clinical Characteristics

The clinical characteristics of the study population (Mean ± SD) including: sex, age, diabetic duration, body mass index, waist/hip ratio, systolic blood pressure and diastolic blood pressure are shown in Table 1.

**Table 1:** Clinical characteristics (Mean ± S.D.) of the study population

	Probability	D.O. (n=29)	D.N.O. (n=29)	N.D.O. (n=15)	N.D.N.O. (n=15)

Sex	0.96	25 (F)/4(M)	25 (F)/4(M)	13 (F)/2(M)	13 (F /2(M)
Age (years)	0.76	48.76 ± 6.91	47.69 ± 9.39	46.47 ± 8.03	46.67 ± 5.51
D.D (years)	0.17	10.38 ± 6.78	8.38 ± 3.77	-	-
BMI (kg/m^2^)	0.00	39.99 ± 8.32	27.22 ± 2.12	42.78 ± 8.33	24.45 ± 2.87
WHR	0.00	0.89 ± 0.01	0.82 ± 0.02	0.89 ± 0.02	0.82 ± 0.01
S.B.P (mm Hg)	0.00	137.24 ± 15.79	131.38 ± 15.75	128.67 ± 9.90	119.33 ± 2.58
D.B.P (mm Hg)	0.02	92.76 ± 22.98	90.69 ± 7.53	85.33 ± 8.34	79.33 ± 2.58

S.D., standard deviation; D.O., diabetic obese; D.N.O., diabetic non-obese; N.D.O., non-diabetic obese; N.D.N.O., non-diabetic non-obese; n, population number; M, male; F, female; D.D., diabetic duration; BMI, body mass index; WHR, waist/hip ratio; S.B.P., systolic blood pressure; D.B.P., diastolic blood pressure.

The sex percentages were kept constant within the groups with a male percentage ranged between (13.3-13.8%) and a female percentage ranged between (86.2-86.7%). No significant differences were found between the groups for all clinical characteristics shown in Table 1.

### Biochemical Changes

The different biochemical markers of the study population (Mean ± SD) including: FBG, HbA1C, Total-C, TAG, HDL-C, ALB, CRT, ALT, AST, CML and sRAGE are shown in Table 2.

**Table 2:** Different biochemical markers (Mean ± SD) measured in the study groups

Parameter	D.O. (n=29)	D.N.O. (n=29)	N.D.O. (n=15)	N.D.N.O. (n=15)

FBG (mg/dl)	267.34 ± 48.16^a^	181.26 ± 41.50	86.48 ± 9.87	74.78 ± 8.62
Hb_A1C_ (%)	10.12 ± 1.30^a^	8.57 ± 1.18	6.28 ± 0.84b	5.11 ± 0.50
Total-C (mg/dl)	242.30 ± 24.55^a^	202.21 ± 26.36	176.09 ± 13.25^b^	147.35 ± 16.85
TAG (mg/dl)	202.27 ± 65.72^a^	152.40 ± 32.80	104.53 ± 19.07	89.81 ± 18.90
HDL-C (mg/dl)	53.87 ± 5.14^a^	61.31 ± 4.73	65.94 ± 3.59^b^	74.76 ± 6.71
LDL-C (mg/dl)	149.28 ± 25.70^a^	110.42 ± 27.09	89.24 ± 14.69^b^	54.21 ± 14.50
ALB (g/dl)	4.67 ± 0.56^a^	5.05 ± 0.54	5.05 ± 0.46	5.29 ± 0.33
CRT (mg/dl)	0.95 ± 0.20^a^	0.85 ± 0.23	0.78 ± 0.13	0.67 ± 0.14
ALT (U/ml)	30.78 ± 9.61^a^	25.18 ± 10.10	24.93 ± 9.26	18.26 ± 6.00
AST (U/ml)	27.21 ± 7.00^a^	20.84 ± 8.97	20.11 ± 4.62	20.12 ± 4.33
CML (ng/ml)	45.40 ± 3.64^a^	36.44 ± 3.23	33.98 ± 3.39^b^	20.70 ± 2.83
sRAGE (pg/ml)	294.68 ± 65.50^a^	333.89 ± 85.82	504.42 ± 112.66^b^	660.60 ± 143.63

D.O., diabetic obese; D.N.O., diabetic non-obese; N.D.O., non-diabetic obese; N.D.N.O., non-diabetic non-obese; n, Number of subjects in each group; FBG, fasting blood glucose; Hb_A1C_, glycated hemoglobin; Total-C, total cholesterol; TAG, triglycerides; HDL-C, high density lipoprotein-cholesterol; LDL-C, low density lipoprotein-cholesterol; ALB, albumin; CRT, creatinine; ALT, alanine aminotransferase activity; AST, aspartate aminotransferase activity; CML, carboxy methyl lysine; Srage, soluble receptor of advanced glycation end product;

a Significant versus Diabetic Non-Obese group;

b Significant versus Non-Diabetic Non-Obese group, significance was set at *P*<0.05.

**FBG and Hb_A1C_.** The D.O group showed a significant increase in both FBG and Hb_A1C_ when compared with those of D.N.O group. The N.D.O group showed a significant increase in HbA1C, but insignificant increase in FBG when compared with those of the N.D.N.O group.

**Lipid Profile.** The D.O group showed a significant increase in serum Total-C, TAG and LDL-C when compared with those of the D.N.O group. Also, the N.D.O group showed a significant increase in Total-C and LDL-C, but an insignificant increase in TAG, when compared with those of the N.D.N.O group. A significant decrease in HDL-C was found in the D.O group compared with the D.N.O group and in the N.D.O compared with N.D.N.O group.

**Albumin.** The D.O group showed a significant decrease in serum ALB when compared with that of the D.N.O group, while no significant relationship was found between ALB of the N.D.O and that of the N.D.N.O group. However, serum albumin in the four studied groups was within the normal range.

**Creatinine.** The D.O group showed a significant increase in serum CRT when compared with that of the D.N.O group. On the other hand, the N.D.O group showed insignificant increase in CRT when compared with that of the N.D.N.O group.

**ALT and AST.** The D.O group showed a significant increase in serum ALT and AST when compared with those of the D.N.O group, while the N.D.O group showed insignificant change in both ALT and AST when compared with those of the N.D.N.O group.

**CML and sRAGE.** The D.O group showed a significant increase in CML and a significant decrease in sRAGE when compared with those of the D.N.O group. Also, the N.D.O group showed a significant increase in CML and a significant decrease in sRAGE when compared with those of the N.D.N.O group.

### Regression Analysis

The whole study samples were divided into two groups: Diabetic and Non-Diabetic to determine the independent predictors of type 2 diabetes. CML (*P*=0.01), sRAGE (*P*=0.00), Hb_A1C_ (*P*=0.00) and FBG (*P*=0.04) were found to be predictors for diabetes (Table 3). The whole study samples were divided into two groups: Obese and Non-Obese to determine the independent predictors of obesity. CML (*P*=0.00), sRAGE (*P*=0.04), Hb_A1C_ (*P*=0.00) were found to be predictors for obesity as shown in Table 3.

**Table 3:** Regression analysis for different biochemical parameters as predictors for Type 2 diabetes and obesity

Parameters	Type 2 Diabetes (n=58)		Obesity (n=30)	
	r	*p*	r	*p*

sRAGE (pg/ml)	-0.33	0.00	-0.13	0.04
CML (ng/ml)	0.29	0.01	0.47	0.00
Hb_A1C_ %	0.43	0.00	0.45	0.00
FBG (mg/dl)	0.22	0.04	0.04	0.76
Total-C (mg/dl)	0.10	0.84	0.26	0.70
TAG (mg/dl)	0.11	0.56	0.03	0.91
HDL-C (mg/dl)	0.01	0.95	0.12	0.79
LDL-C (mg/dl)	0.13	0.79	0.13	0.19
ALB (g/dl)	-0.11	0.06	-0.06	0.43
ALT (U/ml)	0.00	0.98	0.03	0.38
AST (U/ml)	0.00	0.95	0.13	1.72
CRT (mg/dl)	0.02	0.67	0.01	0.89

n, Number of subjects in each group; r, Standardized coefficient; p, probability; sRAGE, soluble receptor of advanced glycation end products; CML, carboxy methyl lysine; Hb_A1C_, glycated hemoglobin; FBG, fasting blood glucose; Total-C, total cholesterol; TAG, triglycerides; HDL-C, high density lipoprotein-cholesterol; LDL-C, low density lipoprotein-cholesterol; ALB, albumin; ALT, alanine aminotransferase activity; AST, aspartate aminotransferase activity; CRT, creatinine.

### Correlation study

**Figure 1:**
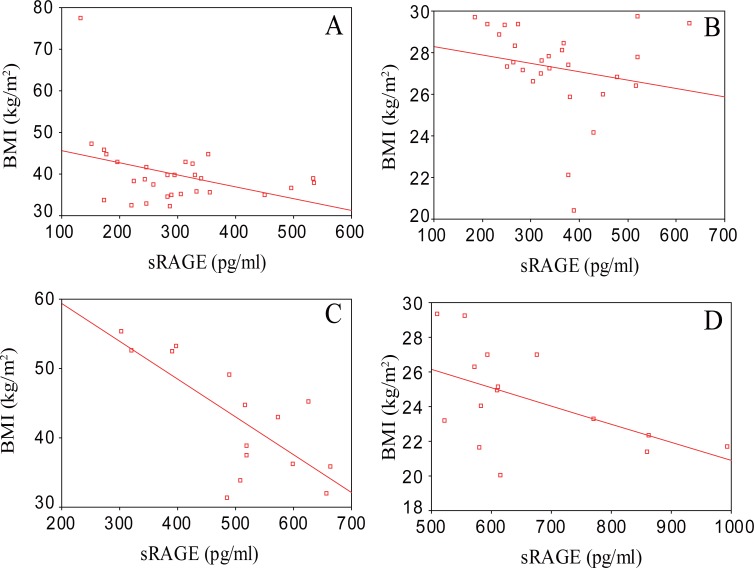
Significant negative correlation between soluble receptors of advanced glycation end products (sRAGE) (pg/ml) and body mass index (BMI) (kg/m^2^) in: **(A)** Diabetic Obese group (r=-0.73, *p*<0.01); **(B)** Diabetic Non-Obese group (r=-0.67, *p*<0.05); **(C)** Non-Diabetic Obese group (r=-0.71, *p*<0.01); **(D)** Non-Diabetic Non-Obese group (r=-0.79, *p*<0.01).

**Figure 2:**
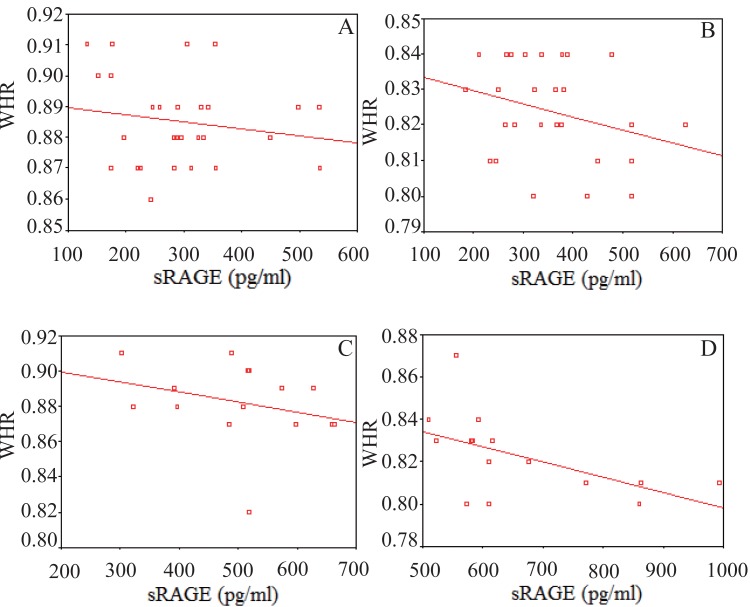
Significant negative correlation between soluble receptors of advanced glycation end products (sRAGE) (pg/ml) and waist/hip ratio (WHR) in: **(A)** Diabetic Obese group (r=-0.81, *p*<0.01); **(B)** Diabetic Non-Obese group (r=-0.73, *p*<0.01); **(C)** Non-Diabetic Obese group (r=-0.52, *p*<0.05); **(D)** Non-Diabetic Non-Obese group (r=-0.63, *p*<0.05).

Pearson correlation coefficient of sRAGE, CML and Hb_A1C_ with different clinical characteristics and biochemical parameters measured in the measured in the four groups are shown in Tables 4-7. sRAGE showed a significant negative correlation with BMI in the four groups with r values of -0.73, -0.67, -0.71 and -0.79 in D.O, D.N.O, N.D.O and N.D.N.O groups, respectively as shown in Figure 1. sRAGE showed a significant negative correlation with WHR in the four groups with r values of -0.81, -0.73, -0.52 and -0.63 in D.O, D.N.O, N.D.O and N.D.N.O groups, respectively as shown in Figure 2. Also, sRAGE showed a significant negative correlation with CML in the four groups with r values of -0.74, -0.57, -0.53 and -0.61 in D.O, D.N.O, N.D.O and N.D.N.O groups, respectively. CML showed a significant positive correlation with BMI in the four groups with r values of 0.52, 0.63, 0.59 and 0.56 in D.O, D.N.O, N.D.O and N.D.N.O groups, respectively.

**Table 4:** Pearson correlation coefficient of soluble receptor of advanced glycation end product (sRAGE), carboxy methyl lysine (CML) and glycated hemoglobin (Hb_A1C_) with different clinical characteristics measured in the D.O. and D.N.O. groups

Parameters	D.O. (n=29)			D.N.O. (n=29)		
	sRAGE (pg/ml)	CML (ng/ml)	Hb_A1C_ (%)	sRAGE (pg/ml)	CML (ng/ml)	Hb_A1C_ (%)

Age (years)	-0.27	0.43^a^	0.65^a^	-0.03	0.61^a^	0.51^a^
BMI (kg/m^2^)	-0.73^b^	0.52^a^	0.81^b^	-0.67^a^	0.63^a^	0.39^a^
WHR	-0.81^b^	0.76^b^	0.61^a^	-0.73^b^	0.48^a^	0.54^a^
S.B.P. (mm Hg)	0.58^a^	-0.018	0.01	0.53^a^	-0.054	0.24
D.B.P. (mm Hg)	0.66^a^	-0.072	0.11	0.51^a^	-0.063	0.28
D.D. (years)	-0.32	0.58^a^	0.31	-0.14	0.49^a^	0.16

D.O., diabetic obese; D.N.O., diabetic non-obese; n, Number of subjects in each group; sRAGE, soluble receptor of advanced glycation end product; CML, carboxy methyl lysine; Hb_A1C_, glycated hemoglobin; BMI, body mass index; WHR, waist hip ratio; S.B.P., systolic blood pressure; D.B.P., diastolic blood pressure; D.D., diabetic duration;

a Correlation is significant at *P*<0.05;

b Correlation is significant at *P*<0.01.

**Table 5:** Pearson Correlation coefficient of soluble receptor of advanced glycation end product (sRAGE), carboxy methyl lysine (CML) and glycated hemoglobin (Hb_A1C_) with different clinical characteristics measured in N.D.O. and N.D.N.O. groups

Parameters	N.D.O. (n=15)			N.D.N.O. (n=15)		
	sRAGE (pg/ml)	CML (ng/ml)	Hb_A1C_ (%)	sRAGE (pg/ml)	CML (ng/ml)	Hb_A1C_ (%)

Age (year)	-0.05	0.42^a^	0.54^a^	-0.01	0.54^a^	0.57^a^
BMI (kg/m^2^)	-0.71^b^	0.59^a^	0.68^a^	-0.79^b^	0.56^a^	0.52^a^
WHR	-0.52^a^	0.49^a^	0.56^a^	-0.63^a^	0.51^a^	0.64^a^
S.B.P (mm Hg)	0.66^a^	-0.27	0.19	0.60^a^	-0.03	0.23
D.B.P (mm Hg)	0.57^a^	-0.06	0.00	0.71^b^	-0.02	0.20

N.D.O., non-diabetic obese; N.D.N.O., non-diabetic non-obese; n, Number of subjects in each group; sRAGE, soluble receptor of advanced glycation end product; CML, carboxy methyl lysine; Hb_A1C_, glycated hemoglobin, BMI, body mass index; WHR, waist hip ratio; S.B.P., systolic blood pressure; D.B.P., diastolic blood pressure;

a Correlation is significant at *P*<0.05;

b Correlation is significant at *P*<0.01.

**Table 6:** Pearson correlation coefficient of soluble receptor of advanced glycation end product (sRAGE), carboxy methyl lysine (CML) and glycated hemoglobin (Hb_A1C_) with different biochemical parameters measured in the D.O. and D.N.O. groups

parameters	D.O. (n=29)			D.N.O. (n=29)		
	sRAGE (pg/ml)	CML (ng/ml)	Hb_A1C_ (%)	sRAGE (pg/ml)	CML (ng/ml)	Hb_A1C_ (%)

sRAGE (pg/ml)	1.00	-0.74^b^	-0.77^b^	1.00	-0.57^a^	-0.52^a^
CML (ng/ml)	-0.74^b^	1.00	0.53^a^	-0.57^a^	1.00	0.39^a^
Hb_A1C_ %	-0.77^b^	0.53^a^	1.00	-0.52^a^	0.61^a^	1.00
FBG (mg/dl)	-0.83^b^	0.51^a^	0.72b	-0.67^a^	0.51^a^	0.73^b^
Total-C (mg/dl)	-0.27	0.20	0.11	-0.27	0.05	0.07
TAG (mg/dl)	-0.23	0.29	0.10	-0.21	0.01	0.30
HDL-C (mg/dl)	-0.01	-0.51^a^	-0.22	-0.05	-0.57^a^	-0.05
LDL-C (mg/dl)	-0.09	0.63	0.27	-0.20	0.54^a^	0.00
ALB (g/dl)	0.04	-0.10	-0.38^a^	0.15	-0.44	-0.41^a^
ALT (U/ml)	-0.04	0.22	0.22	-0.24	0.23	0.20
AST (U/ml)	-0.09	-0.18	0.02	-0.40	-0.24	0.10
CRT (mg/dl)	0.34	0.20	0.04	0.14	0.04	0.12

D.O., diabetic obese; D.N.O., diabetic non-obese; n, Number of subjects in each group; sRAGE, soluble receptor of advanced glycation end products; CML, carboxy methyl lysine; Hb_A1C_, glycated hemoglobin; FBG, fasting blood glucose; Total-C, total cholesterol; TAG, vtriglycerides; HDL-C, high density lipoprotein-cholesterol; LDL-C, low density lipoprotein-cholesterol; ALB, albumin; ALT, alanine aminotransferase activity; AST, aspartate aminotransferase activity; CRT, creatinine;

a Correlation is significant at *P*<0.05;

b Correlation is significant at *P*<0.01.

**Table 7:** Pearson Correlation coefficient of soluble receptor of advanced glycation end product (sRAGE), carboxy methyl lysine (CML) and glycated hemoglobin (Hb_A1C_) with different biochemical parameters measured in the N.D.O. and N.D.N.O. groups

Parameters	N.D.O. (n=15)			N.D.N.O. (n=15)		
	sRAGE (pg/ml)	CML (ng/ml)	Hb_A1C_ (%)	sRAGE (pg/ml)	CML (ng/ml)	Hb_A1C_ (%)

sRAGE (pg/ml)	1.00	-0.53^a^	-0.73^b^	1.00	-0.61^a^	-0.50^a^
CML (ng/ml)	-0.53^a^	1.00	0.55^a^	-0.61^a^	1.00	0.67^a^
Hb_A1C_ %	-0.73^b^	0.56^a^	1.00	-0.50^a^	0.67^a^	1.00
FBG (mg/dl	-0.68^a^	0.66^a^	0.59^a^	-0.69^a^	0.65^a^	0.73^b^
Total-C (mg/dl)	-0.41	0.33	0.18	-0.25	0.08	0.44
TAG (mg/dl)	-0.23	0.20	0.35	-0.27	0.07	0.37
HDL-C (mg/dl)	-0.09	-0.67^a^	-0.30	-0.26	-0.54^a^	-0.03
LDL-C (mg/dl)	-0.33	0.52^a^	0.33	-0.12	0.59^a^	0.37
ALB (g/dl)	0.01	-0.05	-0.49^a^	0.01	-0.20	-0.42^a^
ALT (U/ml)	-0.05	0.36	0.30	-0.09	0.08	0.06
AST (U/ml)	-0.14	-0.16	0.28	-0.06	-0.26	0.12
CRT (mg/dl)	0.00	0.12	0.06	0.21	0.39	0.06

N.D.O., non-diabetic obese; N.D.N.O., non-diabetic non-obese; n, Number of subjects in each group; sRAGE, soluble receptor of advanced glycation end products; CML, carboxy methyl lysine; Hb_A1C_, glycated hemoglobin; FBG, fasting blood glucose; Total-C, total cholesterol; TAG, triglycerides; HDL-C, high density lipoprotein-cholesterol; LDL-C, low density lipoprotein-cholesterol; ALB, albumin; ALT, alanine aminotransferase activity; AST, aspartate aminotransferase activity; CRT, creatinine;

a Correlation is significant at *P*<0.05;

b Correlation is significant at *P*<0.01.

## DISCUSSION

Obesity has been recognized as an important determinant of insulin sensitivity (22). Abdominal obesity plays a central role in the metabolic syndrome and considered a major risk factor for chronic diseases, such as type 2 diabetes and cardiovascular disease. This suggestion is further stressed by the fact that body fat distribution and weight gain throughout adulthood are important predictors of diabetes (23).

In the present study, D.O group showed significant increases in FBG, Total-C, TAG, LDL-C and CRT and significant decreases in HDL-C and ALB when compared with D.N.O group. On the other hand, N.D.O group showed a significant increase in Total-C and LDL-C and a significant decrease in HDL-C when compared with N.D.N.O group. These results are consistent with reports showing that obesity would aggravate type 2 diabetes effects on FBG (24, 25), Total-C (26, 27), TAG (27, 28), LDL-C (25, 27), HDL-C (27, 28), CRT (29, 30) and ALB (29, 31). In addition, our results are consistent with reports indicating that obesity per se would affect Total-C, LDL-C and HDL-C (32).

In the present study, there were significant increases of Hb_A1C_ in D.O group and N.D.O group when compared with those of D.N.O group and N.D.N.O group, respectively.

These results are consistent with reports demonstrating an increase in HbA1C associated with diabetes (33) and obesity (34) suggesting that both diabetes and obesity contribute to the elevation in Hb_A1C_. In diabetes mellitus the extent of the non-enzymatic glycation of proteins increases, compared with non-diabetic subjects, which may comprise at least a part of diabetic complications. Hb_A1C_ provides an integrated measurement of blood glucose during previous 2-3 months, reflecting 120-day life span of erythrocytes (35).

This may explain the significant positive correlation found between Hb_A1C_ and FBG in D.O and D.N.O groups.

Hb_A1C_ elevation associated with obesity may be also attributed to the disordered glucose metabolism. The role of glucose metabolism in Hb_A1C_ elevation may be illustrated by the significant positive correlation between Hb_A1C_ and FBG in N.D.O and N.D.N.O groups and the significant positive correlation between Hb_A1C_ and both BMI and WHR. Hb_A1C_ and BMI correlation is consistent with the findings of Koga and his colleagues (2007), who showed a significant positive correlation between Hb_A1C_ and BMI in 212 non-diabetic normal, overweight and obese subjects (35). Although HbA1C was significantly increased in the N.D.O group versus N.D.N.O group, it remained within the normal range. Moreover, our findings also revealed a significant positive correlation between Hb_A1C_ and age, which are consistent with the previous study of Koga *et al* (2007) (35).

In the present study, there was a significant increase of CML and a significant decrease of sRAGE in D.O and N.D.O groups when compared with those of D.N.O and N.D.N.O groups, respectively. Furthermore, our results showed significant positive correlations between CML and each of BMI, LDL-C and FBG and a significant negative correlation with HDL-C. In addition, sRAGE showed significant negative correlations with CML, BMI and WHR.

Several reports have demonstrated that AGEs are markedly elevated within type 2 diabetic patients and/or illustrated a strong positive correlation between AGEs and BMI. These reports mostly used ELISA kits with polyclonal antibodies showing cross reactivity with many other AGEs or monoclonal ones but specific for AGEs other than CML (36-38). Few reports specifically illustrated CML (using ELISA kits with monoclonal antibodies) and its association with diabetes and BMI (39-41). However, the data still scarce and needs further confirmation. On the other hand, several studies have demonstrated a decline in sRAGE associated with either type 2 diabetes (38, 39) or obesity (21). CML may be considered as a general biomarker for oxidative stress. This could be reasoned by the fact that CML, not only originates from sugars, but also generated from lipids. It is generated *in vitro* from LDL incubated with copper ions and glucose and therefore, is believed to be both lipid and protein adducts. The pathways of CML formation suggest that a direct link of inflammation to AGE formation exists (1).

RAGE was initially identified as a receptor for CML. RAGE–ligand interactions lead to prolonged inflammation, mainly as a result of RAGE-dependent expression of pro-inflammatory cytokines and chemokines (1).

The decrease of sRAGE in type 2 diabetes and obesity has been explained by the beneficial role of sRAGE and its function as decoy to capture the circulating AGEs preventing activation of RAGE signaling pathway as it still possesses the V-ligand essential for ligand binding (1). The decoy function theory is supported by an *in vitro* study, which demonstrated that the treatment with sRAGE normalized the increases in most of the inflammatory markers in diabetic mice to those seen in age matched, non-diabetic controls (40).

Importantly, ligand stimulated RAGE activation results in the generation of reactive oxygen species (ROS), at least in part via the activation of NADPH oxidase. A plethora of evidence suggests that AGEs are involved in a vicious cycle of inflammation, generation of ROS, amplified production of AGEs, enhanced inflammation, and so on. In addition to the ligation of RAGE, AGEs may be linked to increased generation of ROS by multiple mechanisms, such as decreasing activities of superoxide dismutase and catalase, diminishing glutathione stores, and activation of protein kinase C (4). It has been demonstrated that elevated AGEs and advanced lipoxidation end products may participate in the development of vascular disease complications (42). The association between low levels of sRAGE and high levels of CML-protein in patients with microvascular complications suggests that the production of sRAGE was probably insufficient to clear the excess CML-protein. This unbalanced ratio allowed circulating AGE to bind to cell RAGE and activates its signaling pathway (43).

Diet is a major source of exogenous AGEs, as well as, AGEs are especially high in Western diets where foods are processed under elevated temperatures such as by broiling, roasting, deep frying, oven frying, or grilling. In addition, cola drinks, which contain large amounts of caramel additives, are rich sources of AGEs (44). The AGEs content of the same food item can be increased 10-200 fold by increasing the temperature and conditions used in cooking. About 10% of dietary AGEs are absorbed, of which about one third is excreted and two thirds are deposited in tissues (44). Dietary AGEs are implicated in insulin resistance, visceral obesity, and the development of diabetes (36, 45). This may explains the significant positive correlations found in the present work between CML and either BMI or WHR.

The significant positive correlations between CML and either age or diabetic duration may be explained depending upon its role as a general biomarker for oxidative stress. The links between normal physiological ageing and degenerative age-related disease are obvious and there are firm associations between diminished capacity for ageing cells to respond to stress and susceptibility to degenerative disease (46). sRAGE showed a significant negative correlation with CML. The correlation may be explained on the basis of a proposed negative feedback mechanism between CML and serum endogenous soluble receptors of advanced glycation end products (esRAGE) which in turn affect the whole pool of sRAGE. The relationship may include also oxidized LDL whose formation is favored by increased levels of CML and is known to be one of the RAGE ligands (37). In the present work, sRAGE showed a significant negative correlation with systolic and diastolic blood pressure. These results are coincident with some reports which indicated that low sRAGE is associated with hypertension in diabetics and non-diabetics (38, 47, 48).

## CONCLUSION

The present study suggests that non-enzymatic glycation is the pathway connecting diabetes, obesity and diabetic complications including: Hb_A1C_, CML and sRAGE. It indicates that obesity would affect components of the glycation pathway independent of type 2 diabetes. The increase in CML and the decrease in sRAGE associated with obesity found in the present study may support CML role as a general marker of oxidative stress. Also, it supports the decoy function of sRAGE and its beneficial effect in capturing CML. The results may represent a start for more future studies upon the clinical use of sRAGE as an effective medication for various conditions including type 2 diabetes and vascular diseases associated with either type 2 diabetes or obesity. However, the regulation of CML and sRAGE needs further investigation.
